# High-Performance of a Thick-Walled Polyamide Composite Produced by Microcellular Injection Molding

**DOI:** 10.3390/ma14154199

**Published:** 2021-07-27

**Authors:** Dariusz Sykutera, Piotr Czyżewski, Piotr Szewczykowski

**Affiliations:** Department of Manufacturing Techniques, Faculty of Mechanical Engineering, UTP University of Science and Technology, Kaliskiego 7, 85-796 Bydgoszcz, Poland; piotr.czyzewski@utp.edu.pl (P.C.); piotr.szewczykowski@utp.edu.pl (P.S.)

**Keywords:** microcellular injection molding, MuCell^®^ technology, reinforced PA66 GF30 composite, thick-walled moldings, micro CT analysis, FEM simulation

## Abstract

Lightweight moldings obtained by microcellular injection molding (MIM) are of great significance for saving materials and reducing energy consumption. For thick-walled parts, the standard injection molding process brings some defects, including a sink mark, warpage, and high shrinkage. Polyamide 66 (PA66)/glass fiber (GF) thick-walled moldings were prepared by MuCell^®^ technology. The influences of moldings thickness (6 and 8.4 mm) and applied nitrogen pressure (16 and 20 MPa) on the morphology and mechanical properties were studied. Finally, the microcellular structure with a small cell diameter of about 30 μm was confirmed. Despite a significant time reduction of the holding phase (to 0.3 s), high-performance PA66 GF30 foamed moldings without sink marks and warpage were obtained. The excellent strength properties and favorable impact resistance while reducing the weight of thick-walled moldings were achieved. The main reason for the good results of polyamide composite was the orientation of the fibers in the flow direction and the large number of small nitrogen cells in the core and transition zone. The structure gradient was analysed and confirmed with scanning electron microscopy (SEM) images, X-ray micro computed tomography (micro CT) and finite element method (FEM) simulation.

## 1. Introduction

The dynamic development of polymer processing has caused an increase in the use of engineering thermoplastics. To obtain high-quality products with several utility functions, attempts are made to combine various production technologies and their modifications [1,2,3,4]. Key elements of Industry 4.0 in the field of control, monitoring, and optimization of the production process are the important support for obtaining the expected quality of polymeric products [5,6,7]. These supporting tools allow for the qualitative analysis of molded parts in terms of geometric features, surface condition, structure, mechanical properties, and moldings defects in reference to the parameters of the injection molding process.

The mass reduction of structure elements, defined as lightweight parts, while maintaining or improving its mechanical properties is one of the observed trends in product design. In consequence, it is possible to increase the participation of lightweight polymer elements in the construction of cars to reduce their weight, thus reducing fuel consumption and carbon footprint [8,9,10]. Therefore, it can be concluded that Microcellular Injection Molding (MIM) technologies support those activities aimed at producing lightweight products with cells structure for engineering applications [3,11]. Llewelyn et al. reviewed significant scientific achievements in the field of the foamed moldings production using the MIM technology [12]. It can be seen that against the background of the largest number of works with the use of PP, the experimental studies of the MIM with the use of PA66 GF are very poorly represented [13,14]. However, reinforced polyamide material is a popular technical polymer used, among others, in the automotive industry and other important market segments. Unfilled [15,16] or glass fiber reinforced polyolefins [17,18,19], as well as PP blends [15], are usually applied in scientific tests. A wall thickness of examined molded parts with standard and original geometry do not exceed 4 mm in most cases. The relationship between structure and mechanical properties in the aspect of applied MIM process parameters is mainly investigated. Volpe et al. analyzed the effect of melt temperature, nitrogen pressure, and wall thickness (2 3 and 4 mm) on the morphology and mechanical properties of PA66 GF30 foamed molded parts [14]. The authors found that the favorable microcellular structure of PA66 GF30 moldings was obtained by setting the melt temperature at 300 °C. It was found that the moldings’ weight and density decreased with increasing wall thickness and nitrogen pressure. Higher supercritical fluid (SCF) pressure increases the gas content in the melt volume and the cell density in the core region of the molded piece. At the same time, the smallest cells and the largest skin layer were obtained for the smallest analyzed wall thickness of the foamed parts. Cells from 10 to 50 micrometers in size dominate in the core and transition zone. In earlier studies realized by Sykutera et al., the effect of nitrogen parameters in the MuCell^®^ technology on selected mechanical properties and geometric features of moldings were analyzed [20]. The subject was a thin-walled car scuff plate made of PA6 GF30. It was found that an increase in the gas content in the melted polyamide reduces the warpage of foamed moldings by 100% to unfoamed car scuff plates. The weight of the moldings reduced proportionally to the amount of nitrogen, maximal about 10% vol. A decrease in tensile and impact strength and Young’s modulus were observed, at the same time. These changes were not as great as the weight reduced. The observed effect was caused by large cell dimensions (0.067 to 0.098 mm size), which dominated the core and transition zone of foamed parts. An original injection mold and measurement method were applied in the study of the MuCell^®^ technology processability [21,22]. Pressure and temperature sensors were used to online measurement apparent viscosity, shear stress, and a shear rate of melt directly in the injection mold cavity. The conducted tests confirmed the phenomena taking place in the molding cavity for various thermoplastics related to pressure and temperature changes.

The replacement of structural elements made of non-ferrous alloys with hybrid composites is one of the observed trends in technology. It is about reducing CO_2_ emissions and reducing energy consumption. Therefore, it is justified to research in the field of the production of lightweight thick-walled elements from polymeric materials reinforced with short fiber. In the 2020 published paper, extensive results related to the production of thick-walled elements (6 mm and 8.4 mm) with the use of the MuCell^®^ technology were presented [22]. Obtained PA66 GF30 foamed parts characterized the advantageous cellular structure. Small dimensions of cells (about 0.02–0.03 mm) and arrangement of the fibers in the direction of the melt flow were beneficial in terms of potential performance properties. A glass fiber reinforced polyamide 66 was used as an investigated material in this paper. This polymer is of interest for the automotive sector but still little known for high strength thick-wall applications. The own research results so far indicate that it is possible to manufacture cellular, thick-walled elements without the typical defects of thermoplastic molding (shrinkage, warpage). Their wall thickness is much larger than the moldings determined as correctly designed up to the technological rules. This means there is a potential for their use to transmit higher mechanical loads. No similar test results were found. This opens up new areas of potential applications for this type of molding, e.g., in automotive.

The physical properties of the molded parts are formed in the mold cavity and related to the melt flow within the cavity. A typical laminar straight-line flow with a velocity gradient occurs in the area sufficiently distant from the gate. Such a flow determines the orientation of macromolecules and fillers in the core mainly [23,24,25]. The surface effects are the result of the fountain effect at the front of polymer melt and shear in the polymer flow, which is described in the literature [23,24,26]. An appropriate arrangement of the fillers and fibers accompanies the orientation of macromolecules [27,28]. Therefore a multilayer structure of reinforced moldings depends on process parameters, wall thickness, gate type, cooling system and polymer properties and type of fibers. Blowing agents, gas or counter pressure disturbs the fountain effect, change the velocity gradient in the flow front, and therefore varies polymer melt behavior in the skin layer and core zone [28,29,30]. The literature analysis and authors’ own experience show that it is difficult to describe the orientation of the structural elements in porous materials by quantitative indicators [31]. Therefore, the mechanical properties of short fiber reinforced moldings combine with the simulation results are analyzed. However, this applies to the samples of the maximum thickness not exceeding 4 mm, obtained by the conventional injection molding. Quagliato et al. using Autodesk Moldflow Insight/Synergy 2019 found that the shell-core structure, with different fiber orientations and length distribution, significantly impacts the mechanical properties of short carbon fiber reinforced polyamide 6 [32]. Nabiałek et al., one of the few, used the same software to determine cells size distribution and foamed moldings of glass fiber reinforced polypropylene, obtained by MuCell^®^ [33]. Simulation software was also used to evaluate fiber distribution and orientation, weldline reduction, and determine melt viscosity, flow rate, melt and mold temperature, and optimize injection point position [34,35]. MIM process simulation results using Cadmould 3D-F software were not found.

The presented paper aims to study the effect of nitrogen pressure on the structure and selected mechanical properties of foamed thick-walled moldings of PA66 GF30 obtained in the MuCell^®^ technology. The influence of the moldings wall thickness on mechanical properties, like tensile and bending strength, Young’s modulus, impact resistance, and hardness of PA66 GF30 porous composites have been investigated and analyzed. These studies are a continuation of a previously published paper [20,22] on PA66 GF30 thick-walled elements obtained by MuCell^®^ technology. Foamed moldings with no defects were obtained without applying the holding phase. The results of the structural studies obtained in an earlier publication have been extended to explain the behavior of thick-walled, porous PA66 GF30 moldings due to the effects of static and dynamic external loads [22].

## 2. Materials and Methods

**Figure 1:**
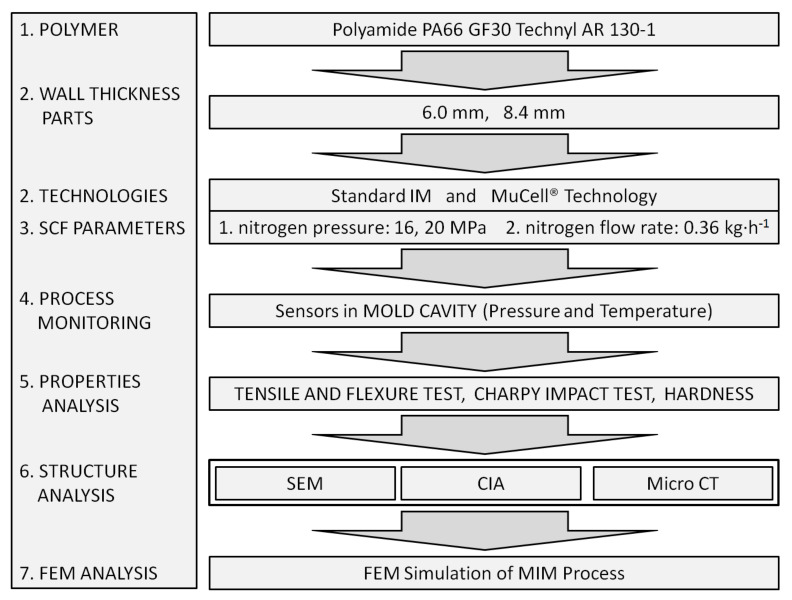
Experiment flow diagram with input and output data.

The experiment flow diagram combining investigated mechanical properties with structural analysis is presented in Figure 1.

### 2.1. Materials

Polyamide PA66 GF30 Technyl AR 130/1 (Rhodia, La Défense, France) reinforced with 30 wt% of short glass fiber (GF) was applied in our experiment. The density of the polymer was 1370 kg·m^−3^, melting point 263 °C, and water absorption 0.8 wt% (at 23 °C, 24 h). The polymer was dried in a vacuum chamber at 80 °C for 4 h, before melt processing.

### 2.2. Testing Specimen and Sample Preparation

#### 2.2.1. Foamed Specimen Preparation

**Figure 2:**
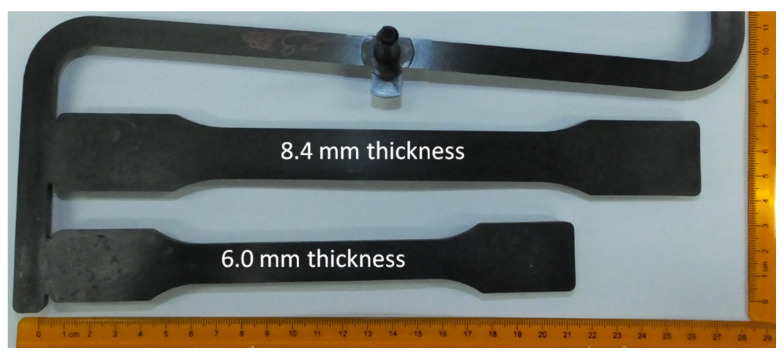
Two thickness types of test specimens produced in one cycle in a balanced injection mold.

**Figure 3:**
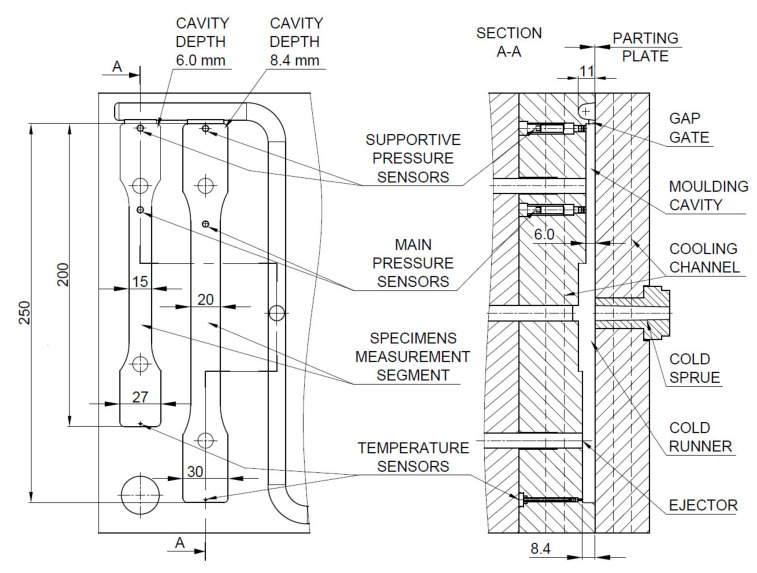
Cavities dimensions and sensors positions inside the special injection mold. All dimensions are in mm.

In the presented study, a four-cavity injection mold was used to produce thick-walled molded pieces. Molded parts dimensions are presented in Figure 2. The injection mold was made of 1.1730 steel with a hardness of 190 HB. The forming plates were made of 1.2312 steel with a hardness of 30 ± 2 HRC and had 290 mm x 235 mm dimensions in the plane of fonts. The mold runner was 183 mm long and the cross-section of 11 mm. 24 mm × 4.2 mm and 21 mm × 4.1 mm (wide × deep) gap gates were applied for thick and thin specimens respectively in the mold cavity. Cooling channels were made symmetrically to the mold parting plane in both molding plates and parallel to the melt flow direction in the mold cavities. The pressure and temperature sensors, type 6002B (sensitive 4.93 PC/bar) and 4008B (thermocouple type N) respectively, were used in each cavity to verify the process reproducibility and moldings quality. The characteristic dimensions of the molding cavities and positioning of the pressure and temperature sensors are presented in Figure 3. The main pressure sensors, located next to the sample measuring area, were used to measure the mold cavity filling conditions. The role of the temperature sensors was to provide a signal when the molding cavities are filled in 99% vol. This determines the switch point from injection to holding and cooling phases. Minimizing holding time up to 0.3 s was necessary due to the injection cycle continuity and did not affect the cellular structure of molding being formed. Assistant pressure sensors (at the gate position) were used to determine pressure changes in the mold cavity during gas injection into the polymer melt. Measurements were recorded during the filling and cooling phases. Received voltage signals were recorded and processed by eDAQ^TM^ 8102 (Priamus, Schaffhausen, Switzerland) transducer (converter).

The foamed moldings were produced by MIM using the MuCell^®^ technology (Trexel, Wilmington, NC, USA). Molded parts were prepared by Engel VC3550/500 Tech (Engel, Schwertberg, Austria) injection molding machine. The processing conditions used during sample production in standard injection molding and MuCell^®^ technology are summarized in Table 1. The parameters of the nitrogen in the supercritical state used in the MIM technology are depicted in Table 2. The method of injection molding and the value of nitrogen pressure were the basis of marking the process and samples with letters A, B, C (Table 2). In our investigations, the N_2_ gas was dosed for 7 seconds, for both pressure values.

#### 2.2.2. Sample Preparation for SEM and micro CT Analysis

**Figure 4:**
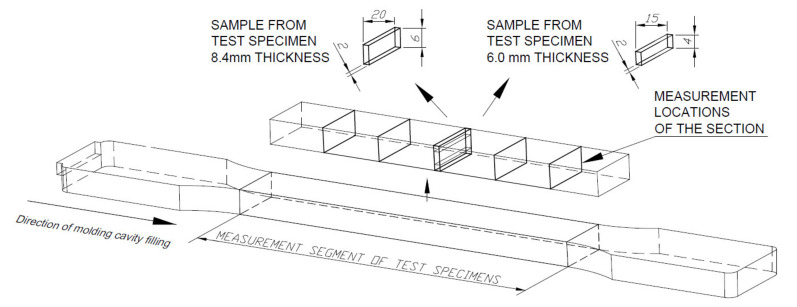
Sampling areas for structural analysis (all dimensions in mm).

The influence of the MIM process parameters on the microcellular structure of PA66 GF30 parts was evaluated by means of scanning electron microscopy (SEM) (JEOL, Tokyo, Japan) and micro computed tomography (CT) (Bruker, Kontich, Belgium) images. The samples for structural analysis were taken from the middle part of the measurement area, as shown in Figure 4.

#### 2.2.3. Sample Preparation for DSC Measurements

**Figure 5:**
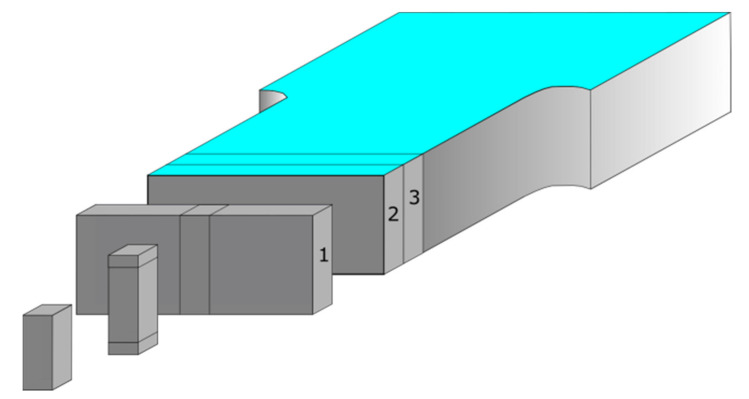
Samples for DSC measurements, schematically presented as the smallest piece in front of the graphic, were taken from three separated stripes (marked as 1, 2, 3) cut in the measurement part of molded piece.

Three stripes were cut from each separate 6 mm and 8.4 mm thick samples A, B and C. The measurement part of molded piece and core parts of 7–12 mg 170 were separated for each differential scanning calorimetry (DSC) test (Netzsch, Selb, Germany) 171 measurements were taken (Figure 5). Average values of the enthalpy of melting of the composite samples were used to calculate the degree of crystallinity.

### 2.3. Measurements of Mechanical Properties

The mechanical properties were determined by tensile and 3-Point bending test using a testing machine Z030 Zwick/Roell (Ulm, Germany) equipped with a measuring head of a nominal load of 30 kN. Conditioned test specimens were cut accordingly to the ISO 527 and ISO 178 from the obtained molded parts. The extension rate was 1 mm/min during the measurements of the elastic modulus for both tests. The next stage of the measurements was carried out at a speed of 50 mm/min (tensile test according to ISO 527) until the samples were broken. Flexural strength was calculated at a speed of 2 mm/min until sample displacement achieved 150% of its thickness (according to ISO 178). The tests were performed at 23 °C for 10 samples from each measurement series. A mechanical extensometer of the type BTC-EXICLEL.001 by Zwick/Roell (Ulm, Germany) was used to measure the sample deformation to determine Young’s modulus as well as strain in the tensile test.

The impact strength of samples was determined using the HIT 50 pendulum impact tester by Zwick/Roell (Germany) with a pendulum of 25 and 50 J. The 25 J pendulum was equipped with a type BPI-ACIPCHI.005 piezoelectric sensor by Zwick/Roell (Ulm, Germany), which enabled the online recording of curves showing changes in the force needed for sample impact break. On their basis, it was possible to determine the impact energy needed to break the tested molded part. A 50 J pendulum was applied for 8 mm thick samples due to their large cross-section. The fracture took place on the shorter edge of the samples. The tests were performed at 23 °C for 10 samples from each measurement series. The hardness was measured by the Brinell method using the 3106 hardness tester by Zwick/Roell. Density was determined by hydrostatic method (methanol as immerse liquid) with the use of AD50 (Axis Sp. z o.o., Gdańsk, Poland) laboratory scales.

### 2.4. SEM and Micro CT Analysis

Sample surface was investigated with a JEOL 5600 electron microscope (JEOL, Tokyo, Japan) at 1 kV acceleration voltage, after sputter coating with platinum layer. Scanning electron microscopy (SEM) samples were cut, a notch was made on both sides of a sample. Notch was made along on the bigger surface, merged in a liquid nitrogen and broke with a hammer. Each sample was scanned from left to right edge and pictures of 150× magnification were joined together in order to estimate the thickness of skin, transition and core layer.

Sample C8 was investigated by SkyScan 1272 X-ray micro computed tomography (Bruker, Kontich, Belgium) with an image pixel size of 0.35 µm, by a source voltage of 70 kV. Projections were reconstructed by the NRecon program, the analysis was done using the CT Analyzer program, while 3D visualization of glass fiber orientation was obtained by CTvox program. Sample of c.a. 1 mm^3^ size in volume was cut.

### 2.5. DSC Measurements

Differential scanning calorimetry (DSC) was used to determine differences in crystallinity level in a core part of polyamide matrix with and without pores by using DSC 214 Polyma apparatus by Netzsch. Samples were heated up to 310 °C in a nitrogen atmosphere, cooled down to 25 °C after two minutes, and heated again at a cooling/heating rate 10 K/min.

The degree of crystallinity α was estimated based on the enthalpy of melting of PA66 composite samples ∆H_m_, and the enthalpy of melting of a 100% crystalline PA66 ∆H_0_, accordingly to the equation:

(1) $$\alpha =\frac{\Delta H_{m}}{\Delta H_{0}\left(1-\varphi \right)}100\%$$

where, φ is the filler content assumed as 0.3 weight fraction accordingly to the producer. The enthalpy of melting of 100% crystalline PA66 was taken as 188 J/g [36].

### 2.6. FEM Simulation of MIM Process

Cadmould 3D-F software ver. 13.0.2.1. by Simcon Kunststofftechnische Software GmbH (Würselen, Germany) was used to simulate MIM process. The Fine Element Method (FEM) volumetric model was applied in the simulation, uniform for the cavities, runners, and gates. Virtual sensors positioning was adopted based on the location of the sensors in the laboratory mold (Figure 2). Table 3 summarizes the parameters used to prepare the FEM model of a cavity.

Simulation analyses were performed using the same foamed process parameters and the same type of reinforced polyamide as in the laboratory experiment. Nitrogen pressure of 20 MPa was set (example C).

## 3. Results and Analysis

**Figure 6:**
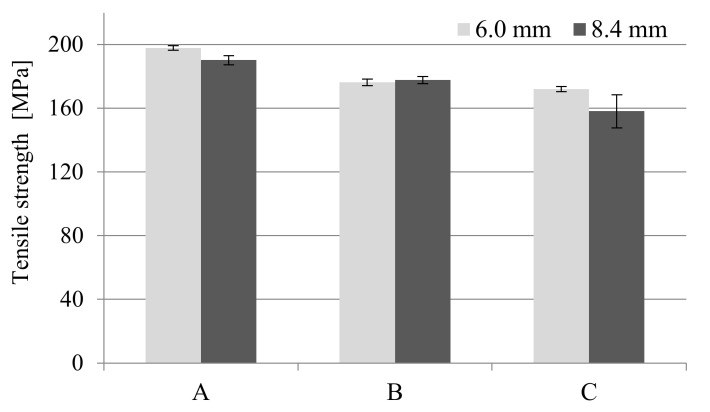
Influence of nitrogen pressure on the tensile strength for unfoamed and foamed thick-walled PA66 GF30 molded parts, where A is a solid sample, while B and C are foamed at 16 and 20 MPa nitrogen pressure respectively.

**Figure 7:**
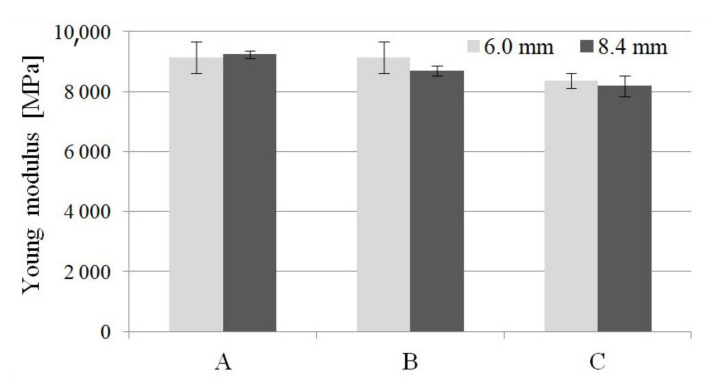
Influence nitrogen pressure on the Young modulus for unfoamed and foamed thick-walled PA66 GF30 molded parts, where A is a solid sample, while B and C are foamed at 16 and 20 MPa nitrogen pressure respectively.

The values of stress and Young’s modulus (during the tensile strength test) for foamed molded parts obtained in MuCell^®^ technology slightly decreased compared to unfoamed samples (Figure 6 and Figure 7). The greatest changes were observed for samples C regardless of the thickness of the sample. This means that the nitrogen pressure has a significant influence on the formation of gas pores and more of them were formed in the samples of type C. Despite the noticed changes, it can be concluded that the thick-walled samples B and C have very good strength properties, even higher than those provided by the material manufacturer (9200 MPa and 6000 MPa for dried and conditioned samples, respectively).

A similar effect can be observed during the tensile strength tests. One can find values of about 140 MPa and 100 MPa tensile strength in the product data sheet for unfoamed, dried and conditioned samples PA66 GF30, respectively. The manufacturer of this material most probably provided the value for samples with a smaller thickness, in accordance with the ISO 527 standard (4 mm sample thickness). Although the analyzed molded parts are thicker (6 and 8.4 mm), which is not recommended for technological reasons, very high tensile strength values were obtained, higher than solid samples provided by the manufacturer.

**Figure 8:**
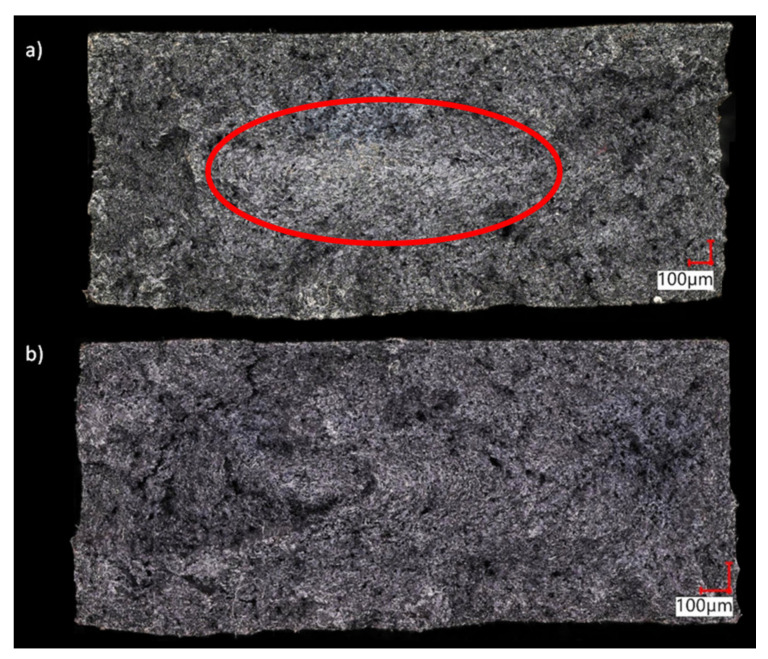
The fractures of the PA66 GF30 samples with a thickness of 8.4 mm obtained in the static tensile test: (**a**) unfoamed sample A, (**b**) sample C foamed with nitrogen at the pressure of 20 MPa.

Figure 8 shows examples of fractures of non-foamed and foamed samples (Figure 8a,b respectively) obtained in the static tensile test. The mean relative deformation for these samples ranged from 4.92 to 5.90% for samples A and C, respectively. With 6 mm thick samples, the range of changes of this parameter was within a narrow range from 5.98% (sample A) to 5.60% (sample C). As expected, the addition of glass fibers reduced the deformability of the polyamide matrix and the disappearance of the neck effect for all samples. It is for this reason that brittle fractures were obtained, while for non-porous samples, the whitening effect can be noticed over a large fracture area (in the core and partially in the transition zone), which indicates a partial deformation of the macromolecules toward the tensile force of a plastic nature. This is consistent with literature reports indicating reinforced fibers arranged perpendicular to the direction of flow in the core of samples. This effect is more intensively observed the greater the thickness of the fiber-reinforced samples.

For all porous samples, the fractures are free of whitening zones (Figure 8b). The significant thickness of the test objects and a significant reduction in the apparent viscosity of the melt in the MIM process [22] reduced the pressure drop and velocity gradient at the melt front. This in effect changed the fiber orientation behind the melt front. This is a similar effect to the influence of gas counter pressure and mold temperature on the fountain flow effect described in the literature [23,29,30]. In MIM technology, both these effects are reduced, which affects the orientation of the fibers throughout the cross-section.

Additionally, the sample geometry and the shape of the gate enabled a laminar flow with a small velocity gradient, as evidenced by the values of shear stresses presented in the previous article [22]. Despite the laminar flow, the fountain effect was visible through the silvering of the sample’s surface.

**Figure 9:**
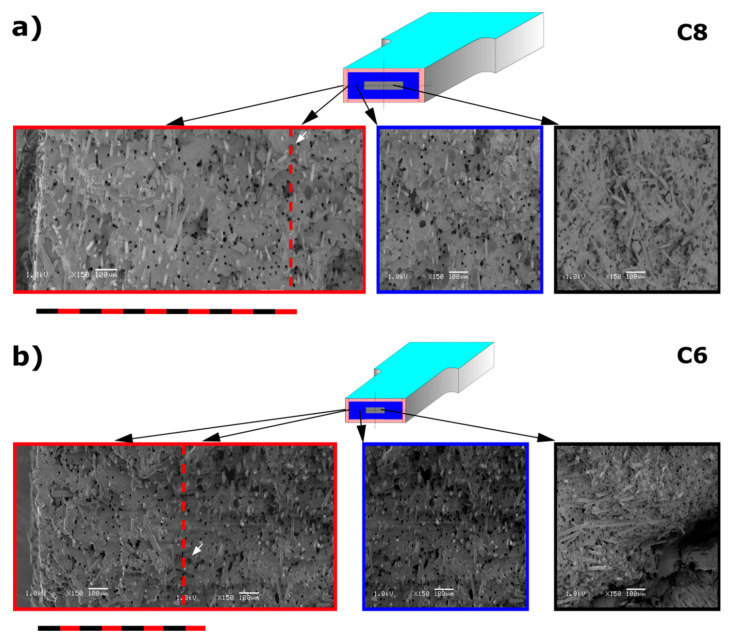
Scanning electron microscopy pictures taken from different regions of measurement parts of 8 mm (**a**) and 6 mm (**b**) thick molded pieces. SEM pictures on left (red frame) present a sample edge with the assumed border of a skin layer marked with a dashed line. The white arrow indicates the first observed pore, suggesting the end of a skin layer. Pictures in the middle (blue frame) present the transition layer while pictures on the right (black frame) show sample core.

This proves the desired fiber arrangement (in the flow direction) in the skin and the transition zone. These areas are very thick, as shown in Figure 9. It seems that one of the reasons for this phenomenon was the appropriate design of the slot gate (with a large cross-section, adapted to the size of the samples). Homogeneous morphology of thin-walled parts was presented in our earlier paper and documented by further SEM tests [22]. This wide zone of the fibers parallel positioned to the direction of melt flow determines such high values of tensile strength for all thick-walled samples. This is confirmed by the SEM images (Figure 9).

Presented results show an increase in areas where the short fibers are arranged in the flow direction, which is in line with the direction of the tensile force. As a result, the obtained composites transfer tensile loads in a better way. Here one can recall the influence of the arrangement of the continuous fiber in composites with cross-linked resin matrices. Using unidirectional (UD) tapes toward the external load is preferable for such structures. Representative results of SEM pictures are presented in Figure 9 for 8.4 mm (C8) and 6 mm (C6) thick samples. Pictures on the left (in red frame) represent the outer, skin layer of the sample. By the red dashed line the assumed end of a skin layer is marked, based on the first observed pore indicated by the white arrow. This way the skin layer thickness is assumed as 1200 µm and 700 µm for samples C8 and C6 respectively. It can be observed, that glass fibers are mainly oriented in a polymer melt flow direction in a skin layer since fibers stick out perpendicularly to the cross-section of the sample and there are basically no fiber oriented in parallel to the surface. There is a higher amount of empty holes (deep black spots of c.a. 9 µm diameter) left after glass fiber removing during a break comparing to the inner region of a sample, which additionally indicates glass fiber orientation parallel to the melt flow direction. The picture in the middle (blue frame) represents the transition phase with clearly visible pores and partly randomly distributed glass fibers. The picture on the right (black frame) represents the core of the sample. Much more disorder, fibers perpendicular to the flow direction and big gaps in material (e.g., sample C6 in Figure 9b in a right bottom corner) are visible in this region. Observed lack of material is caused by breaking the sample and its weakness in the core part comparing to the outer layer. Those pictures confirm the outer shell and transition zone built from oriented glass fibers responsible for sample strength. Based on the analysis of SEM pictures the thickness of skin, transition and core layer was estimated for samples C8 and C6. The core part is about 48% and 30% for samples C8 and C6, respectively (Table 4). These observations are in agreement with other papers [12,13,14,23,24].

**Figure 10:**
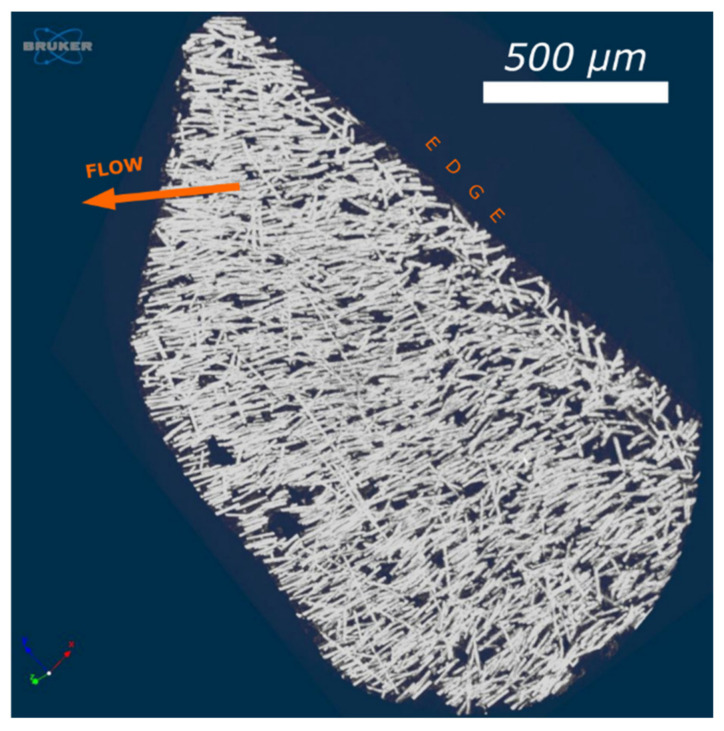
Microcomputed tomography 3D visualization of glass fibers orientation in a sample cut from the edge of 8.4 mm thick molded piece (C8). Polymer melt flow is marked by the orange arrow.

Fiber orientation in the flow direction was confirmed by high-resolution micro-CT scans as well. The 3D visualization presented in Figure 10 shows fibers orientation into c.a. less than 1 µm deep. It confirms an existing of a skin layer within 1 µm from the edge of C8 sample as observed by scanning electron microscopy. Further analysis of pore size distribution and glass fibers orientation within the sample cross-section will be presented in the separate article.

**Figure 11:**
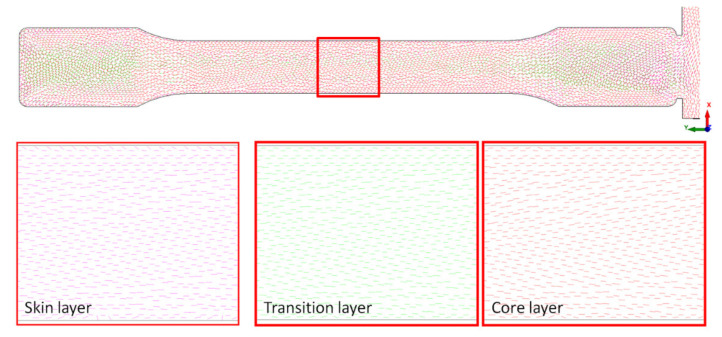
Simulation of the fiber orientation in the center of a measuring part (marked with a red square) for a skin, transition and core layer in a porous sample C8.

Additionally, to previous images, FEM confirmed the fiber orientation in the melt flow direction in the transition zone and skin layer (Figure 11). Those structural and simulation analyses are an unquestionable proof of the satisfying mechanical properties of foamed PA66 GF30 parts.

**Figure 12:**
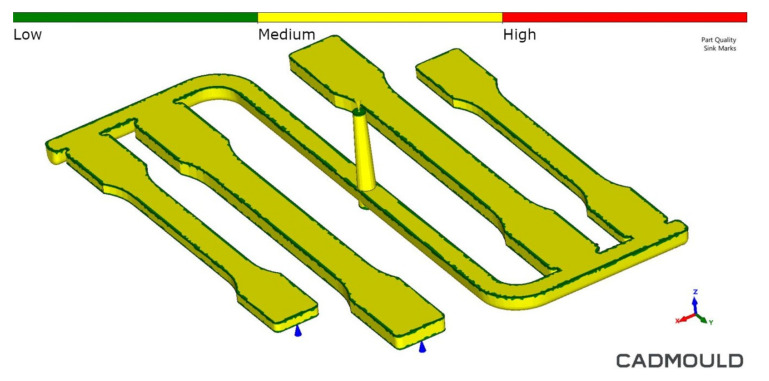
Simulated final sink marks distribution on the foamed PA66 GF30 parts surface.

The simulation results also confirmed the good surface quality and shrinkage minimization of real samples obtained in the MIM technology. No significant sink marks (red color in the scale) were found on the thick-walled moldings (Figure 12). When using the Sink Marks function, the yellow color indicates potential areas of this surface defect, but it does not have to occur in a real injection molding process.

Based on DSC measurements of surface area under the enthalpy of melting peaks, the differences are too small to confirm the influence of physical porosity on the crystallinity level. The average values of the melting enthalpy of the PA66 composite ΔH_m_, melting temperature T_m_, sample mass, and calculated crystallinity level α are presented in Table 5. On this basis, it can be concluded that the degree of crystallinity did not affect values of mechanical properties.

**Figure 13:**
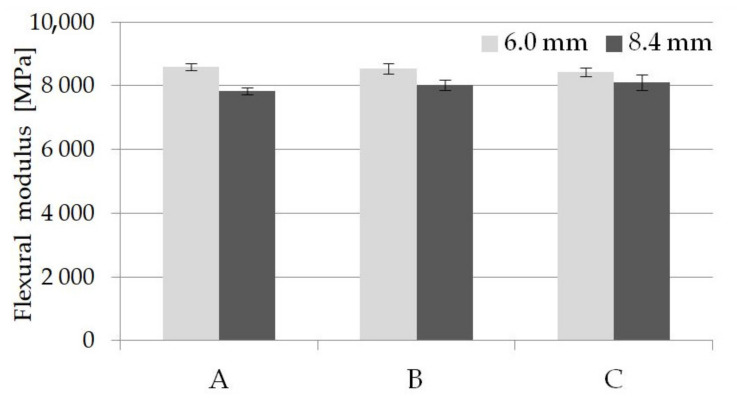
Influence of nitrogen pressure on the flexural modulus for unfoamed and foamed thick-walled PA66 GF30 molded parts, where A is a solid sample, while B and C are foamed at 16 and 20 MPa nitrogen pressure respectively.

**Figure 14:**
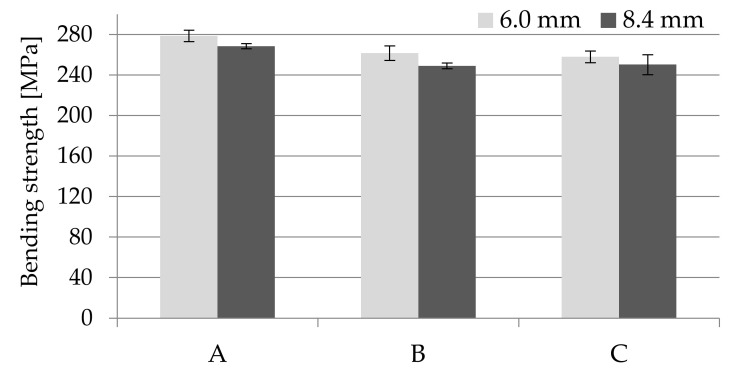
Influence of nitrogen pressure on the bending strength for unfoamed and foamed thick-walled PA66 GF30 molded parts, where A is a solid sample, while B and C are foamed at 16 and 20 MPa nitrogen pressure respectively.

The bending test results confirmed the importance of the transition zone in the walled-thick samples. Figure 13 shows the flexural modulus of unformed and foamed PA66 GF30 molded parts. It can be observed that the bending modulus of foamed 8.4 mm thick samples has higher values comparing to unfoamed and this difference increases with the nitrogen pressure. For samples with a thickness of 6 mm, the modulus changes are like those in the tensile test. The highest values obtained for unfoamed samples were higher than those stated by the plastic supplier in the product sheet (8250 MPa for dried PA66 GF30 molded parts). It seems that maintaining such a high stiffness of foamed parts is due to the orientation of the glass fibers in the direction of melt flow in the skin and transition layer. The effect of both sample thickness and the use of supercritical nitrogen in the injection molding process on the bending strength is similar to that changes in the tensile test (comp. Figure 6 and Figure 14). Comparison of the bending strength with the manufacturer’s data shows that all tested samples had a higher bending strength than those given in the datasheet (227 MPa for dry unfoamed PA66 GF30 samples). Importantly, the application of foamed technology did not cause a significant decrease in bending strength for both types of foamed (B and C) samples. These average values are superior comparing with stated by the manufacturer for solid PA66 GF30. Homogeneous porous structure and the tiny cell dimensions, especially in the core, are one reason for a such behavior of foamed samples in the bending test (besides the orientation of the fibers) [20].

**Figure 15:**
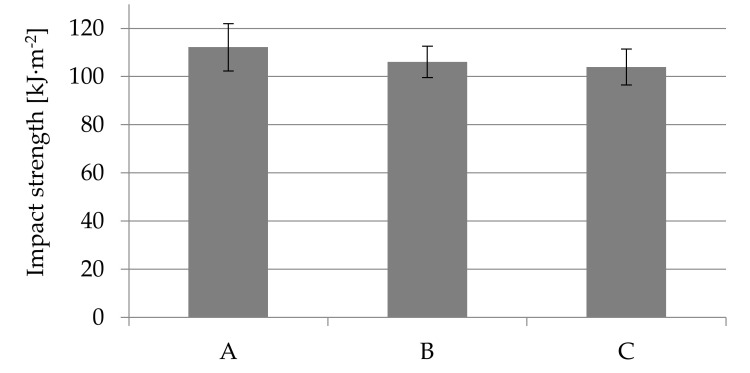
Influence of nitrogen pressure on the impact strength for unnotched thick-walled PA66 GF30 moldings with a thickness of 6 mm, where A is a solid sample, while B and C are foamed at 16 and 20 MPa nitrogen pressure respectively.

Comparing to the data of the material supplier and the Campus database, the obtained impact toughness results are improved for all samples. The Charpy unnotched impact strength was 112.1 ± 9.8 kJ·m^−2^ for solid molded parts with a thickness of 6 mm, which is over 70% higher than the supplier’s data (65 kJ·m^−2^ for conditioned samples). Foaming of the PA66 GF30 samples reduced the impact toughness, but only to the values of 106.1 ± 6.5 and 104.0 ± 7.5 kJ·m^−2^, for samples B and C respectively (Figure 15).

It seems that the reason for such good impact results is their homogeneous porous morphology and very small cells dimensions, especially in the core, as well as the similar orientation of the glass fibers both in the skin and in the thick transition layer (Figure 9, Figure 10 and Figure 11).

**Figure 16:**
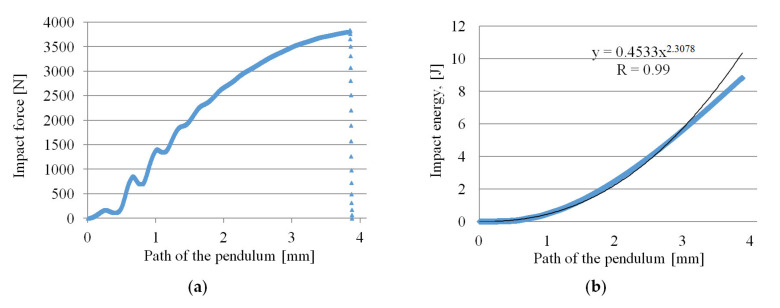

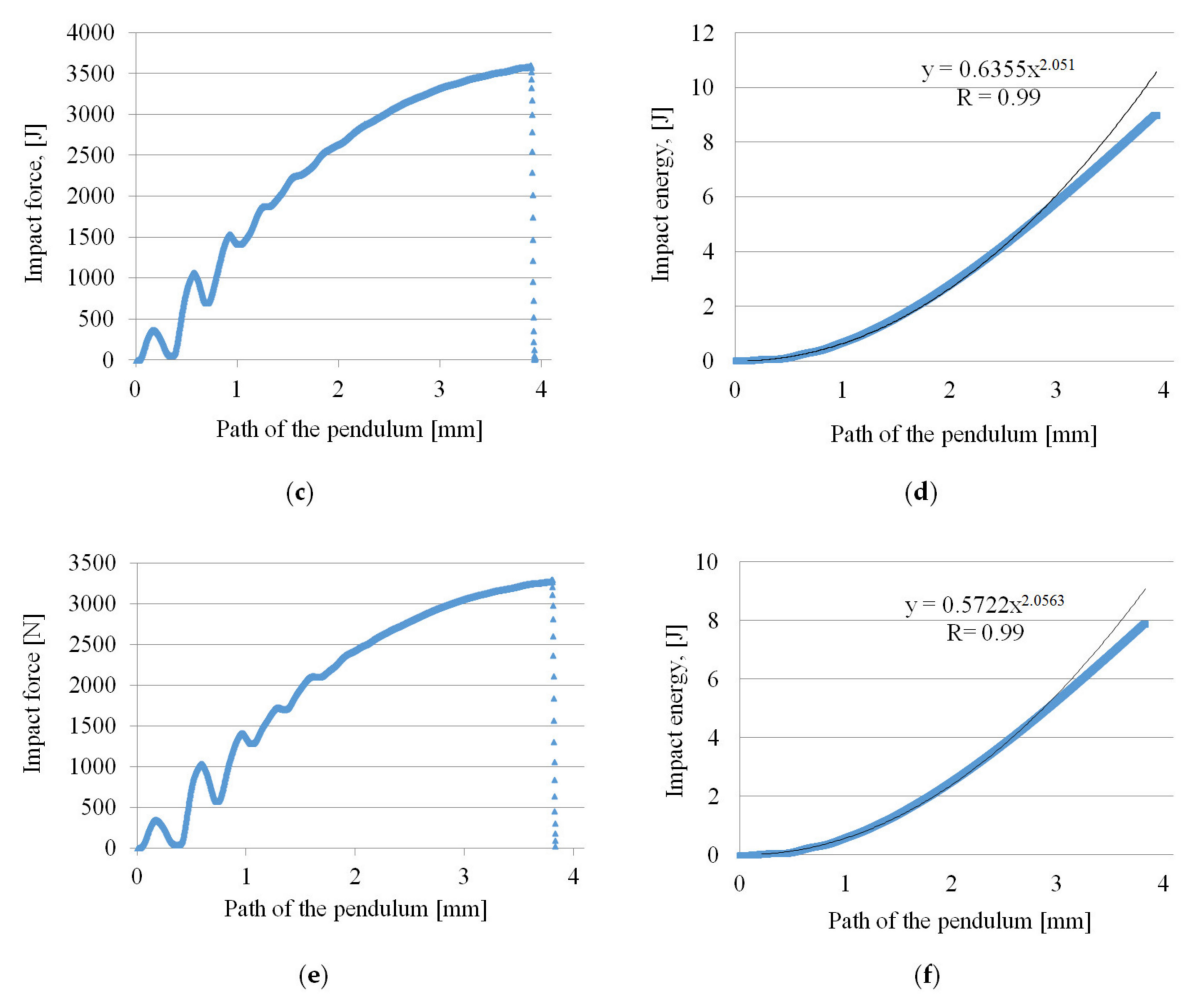
Courses of the impact force and the cumulative impact energy during the impact tests of PA66 GF30 samples with a thickness of 6 mm: (**a**,**b**) unfoamed moldings A, (**c**,**d**) foamed moldings B, (**e**,**f**) foamed moldings C.

The analysis of the impact force curves shows that the only differences are the reduction of their maximum value and the shorter displacement of the pendulum until the specimens were completely impact fractured (Figure 16). For the samples obtained using the highest nitrogen pressure (marked C), both parameters influencing the impact energy are the lowest. This effect is because the pores are the largest for samples C, especially in the core zone (see Figure 9).

**Figure 17:**
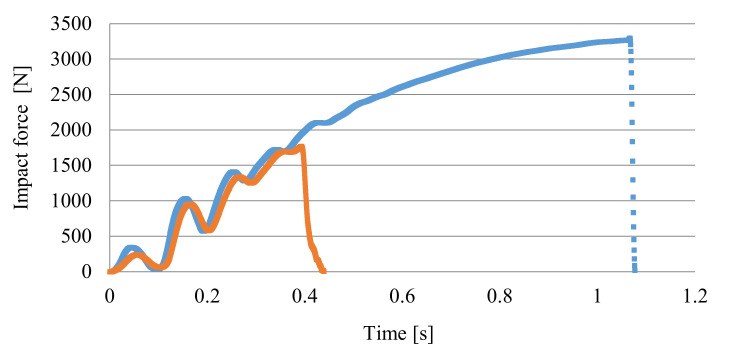
Courses the impact force and during the impact tests of PA66 GF30 unnotched (blue line) and notched (orange line) samples C with a thickness of 6 mm.

At the same time, it can be observed that a power function can describe the cumulative impact energy curves. For 6 mm-thick unnotched samples, the function exponent is 2.3, and for foamed samples B and C it is smaller and amounts to 2.05. This indicates a more intense increase in impact energy of A (unfoamed) samples needed for their fracture. A notch with a depth of 1 mm on sample C (a value close to the thickness of the skin) causes a significant decrease in the impact strength to the value of 17.03 kJ·m^−2^, which is only 16.4% of the impact toughness for unnotched samples (104.0 kJ·m^−2^). A notched samples break at twice less maximum impact force in a time shorter more than twice, compared to unnotched samples (Figure 17).

**Figure 18:**
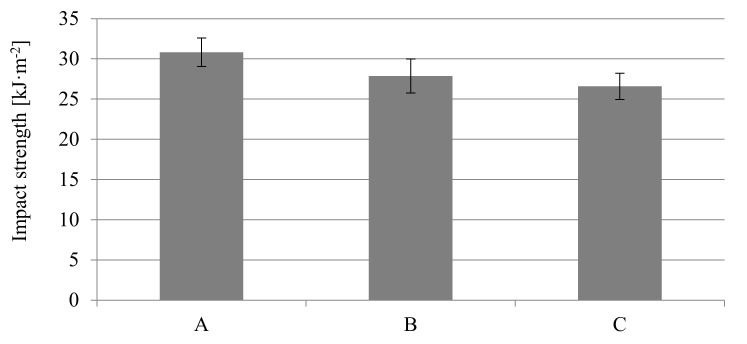
Influence of nitrogen pressure on the impact strength for notched PA66 GF30 moldings with a thickness of 8.4 mm, where A is a solid sample, while B and C are foamed at 16 and 20 MPa nitrogen pressure respectively.

The impact strength of notched 8.4 mm thick samples is much higher than for notched specimens with a thickness of 6 mm (Figure 18). The value of this parameter slightly decreased for foamed samples.

**Figure 19:**
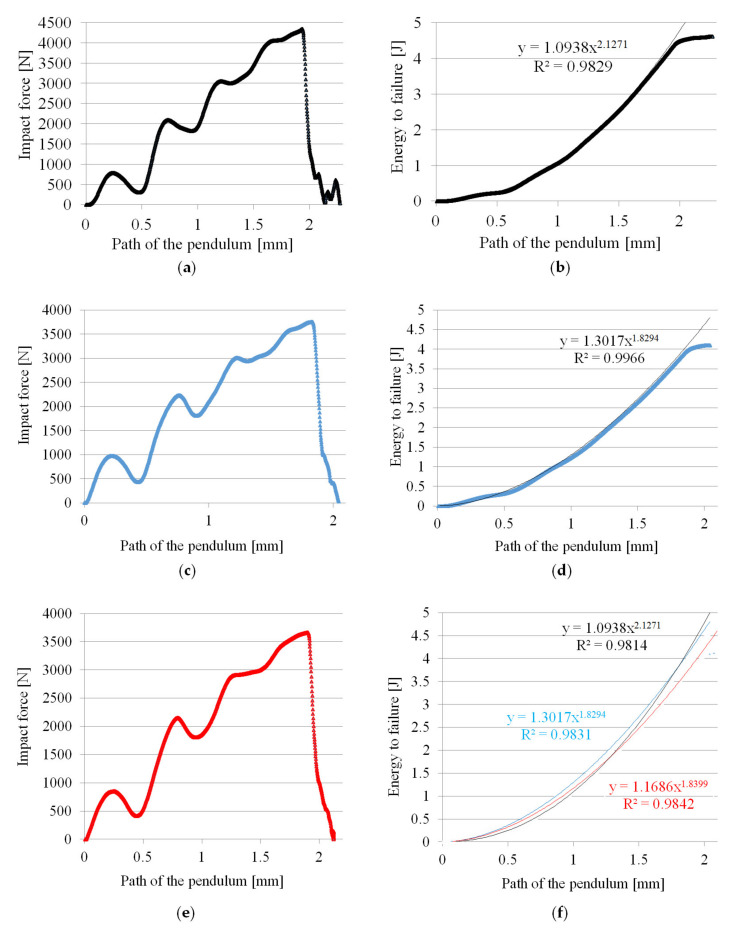
Courses of the impact force and the cumulative energy of failure during the impact tests of PA66 GF30 samples with a thickness of 8.4 mm: (**a**,**b**) unfoamed moldings A, (**c**,**d**) foamed moldings B, (**e**,**f**) foamed moldings C.

Curve courses of changes in the impact strength and cumulative impact energy are similar to those described earlier for 6 mm-thick samples (compare Figure 16 and Figure 19). However, these graphs cannot be compared, because those 8.4 mm-thick samples had a 1 mm-thick notch. It should be noticed that despite this, the values of the impact strength are at a very good level. This appears to be due to a very thick transition layer and a favorable earlier showed orientation of the fibers. This appears to be due to a very thick transition layer and a favorable fibers orientation, which was shown earlier. A power function can describe the cumulative impact energy curves in this case as well. The function exponent is 2.13 for 8.4 mm-thick notched samples A, and amounts to lower 1.83 and 1.84 values for foamed samples B and C, respectively. Curves are similar in shape for all samples.

It can also be observed that the samples fractured after the pendulum displacement of about 2 mm, while the cracking time was only 0.6 s. It can be stated that both the increase of PA66 GF30 moldings thickness up to 6 and 8.4 mm value, as well as the foamed structure, did not significantly reduce its toughness compared to the impact strength results for less thick molded parts. It was possible thanks to the multilayer structure of molded parts, favorable orientation of the glass fibers in the skin and the transition zone, and the small size of the gas cells in the moldings structure.

**Figure 20:**
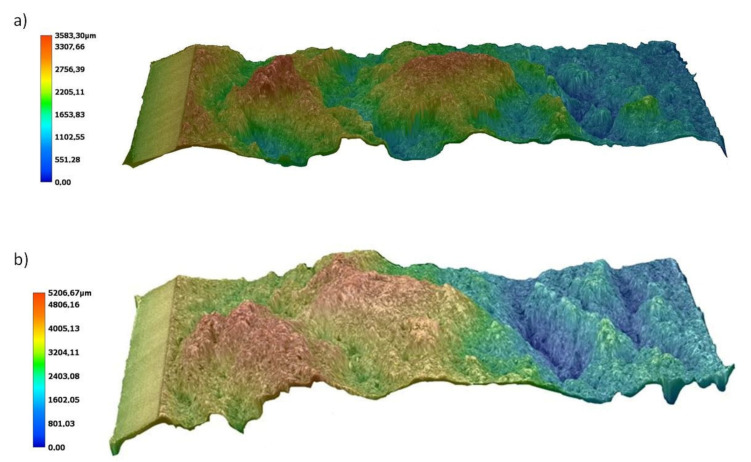
Topography of fractures of samples with a thickness of 8.4 mm obtained in impact tests: (**a**) unfoamed sample A8, (**b**) sample C8 foamed with 20 MPa nitrogen pressure.

The effect of differentiating the arrangement of the fibers in the skin (along the flow direction) and in the core (perpendicular to the flow direction) can be observed in the images of the fractures of solid and porous samples, Figure 20a,b respectively. The hammer hitting the sample acts in the fiber’s direction alignment in the core zone, which causes a significant part of the reinforcement to be sheared and compressed and therefore susceptible to deformation. This results in smaller variations in the breakthrough topography (see Figure 20a). With the porous structure of samples B and C, the less oriented fiber structure in the core part and the gas pores promote better transfer and damping of the shock load. This translates into the observed variation in the topography of the obtained fractures (see Figure 20b). The difference between the highest and lowest points of the fracture surfaces is the lowest for the solid sample A and increases with increasing nitrogen pressure, even by about 45% for the samples C (Figure 20b). The significant thickness of the transition zone with the proper glass fibers orientation and the small dimensions of the cells, also in the core, is very important for the observed effects.

**Figure 21:**
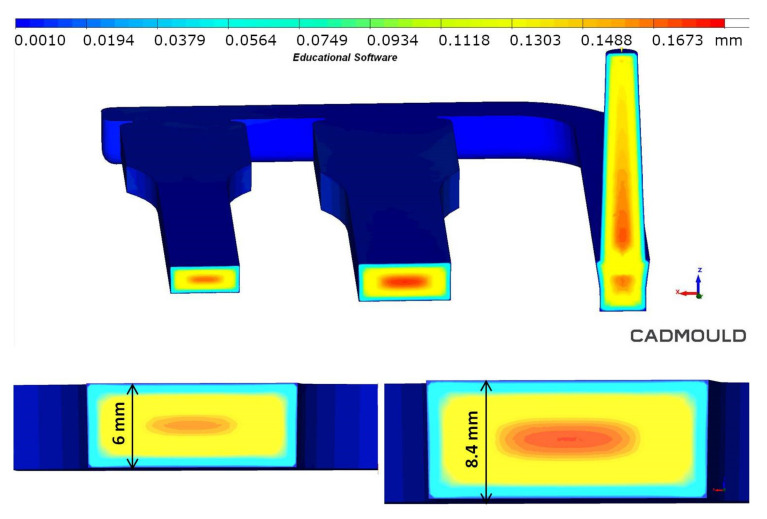
Simulated pore size distribution in the cross-section of 6 mm and 8.4 mm thick polyamide test specimens.

The pore size distribution in the foamed molding type C was confirmed by simulation. However, it should be noted that simulation graphs revealed much larger pores in transition and core zone comparing to SEM pictures of real moldings (Figure 9). The pore size and distribution shown in the graph indicate that the simulation program assumes cell growth until the end of the cooling phase (Figure 21). The reason for the discrepancy in the results may be the FEM method and/or algorithm used for the simulation of MIM, which does not work well in the calculation of thick-walled foamed elements (6.0 and 8.4 mm).

**Figure 22:**
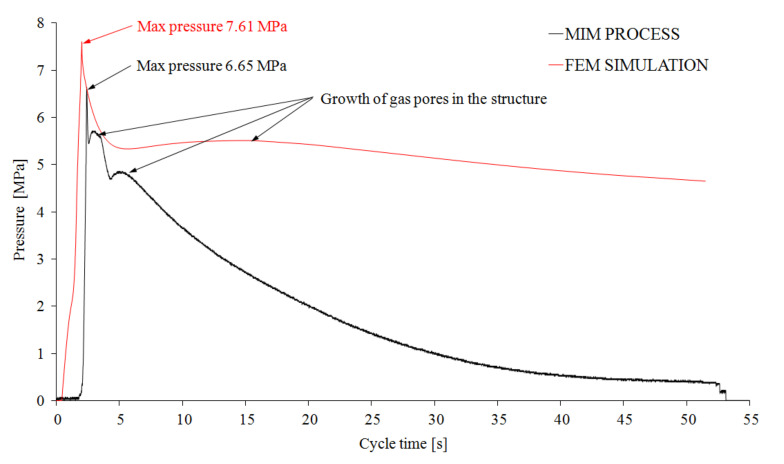
Pressure changes in the molding cavity for real (MIM) and simulated (FEM) processes, black and red curves, respectively.

This continuous growth of pores noted for the simulation can also be indirectly observed in the graphs of pressure changes in the injection mold (Figure 22). The curve obtained in the simulation test (red color in Figure 22), also after the cooling phase, shows the pore growth effect. According to the authors, this noticeable difference in the pressure’s course curves results from the surface mesh finite element model used in the Cadmould 3D-F program. The significant thickness of the analyzed object (8.4 mm) influenced the obtained results as well.

The changes in injection pressure in the injection mold cavity registered during the realization of technological tests are characteristic of MIM and different from the standard injection molding process. The pressure changes (Figure 22) showed in the diagram confirm the effects described in the previous papers [22]. The maximum pressure value in the cavity is low and does not exceed 7 MPa. The rapid drop in pressure in the mold cavity after the injection and hold-down phases (0.3 s) was slowed down by cell growth, especially in the core part, and was continued until the end of cooling phase. The observed phenomena are influenced by the very large sample thickness (8.4 mm). Registered changes confirm the results of simulation tests (Figure 22). The displacement of the pressure curves registered in the mold cavity was observed (Figure 22). It was caused by the additional work steps of the injection molding machine in the real process. The showed difference in maximum cavity pressure between the runs is only 1 MPa. It can therefore be concluded that obtained multi-layer polyamide structures dampen the impact energies of the pendulum in a good way.

**Figure 23:**
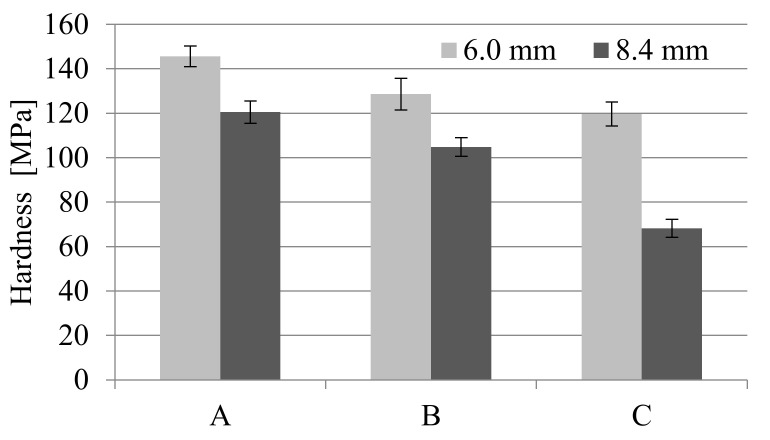
Influence of MuCell^®^ parameters and moldings thickness on the hardness, where A is a solid sample, while B and C are foamed at 16 and 20 MPa nitrogen pressure respectively.

A significant influence of the thickness of the moldings on their hardness was found. Nitrogen pressure significantly influenced this property as well (Figure 23). For all 8.4 mm thick samples, the hardness is lower compared to those with a thickness of 6 mm. Microcellular injection molding with the use of a gas pressure of 20 MPa caused a twice decrease in the hardness of these samples compared to solid moldings. In the case of foamed samples with a thickness of 6 mm, similar effects were obtained, but on a smaller scale. The decrease in hardness for C6 samples compared to A6 was only 17.8%. That means that the 5 mm diameter metal ball penetrated the sample’s porous structure more easily during the hardness test.

**Figure 24:**
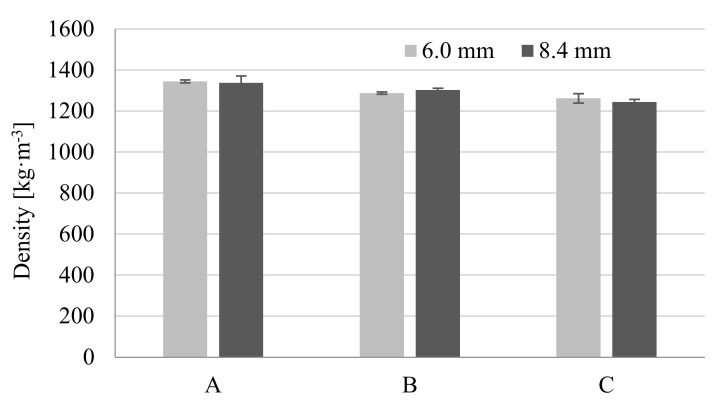
Influence of MuCell^®^ parameters and moldings thickness on the density of molded parts, where A is a solid sample, while B and C are foamed at 16 and 20 MPa nitrogen pressure respectively.

Observed changes in the moldings density are the indirect evidence explaining this phenomenon (Table 6 and Figure 24). Density decrease was observed for foamed moldings compared with solid ones, regardless of the sample thickness. The largest reduction was recorded for samples C by 6.16% and 7.03% for 6.0 mm and 8.4 mm thickness, respectively. This can be explained by the increase in core zone for 8.4 mm-thick moldings and easier penetration of higher pressure gas in the polyamide melt.

**Figure 25:**
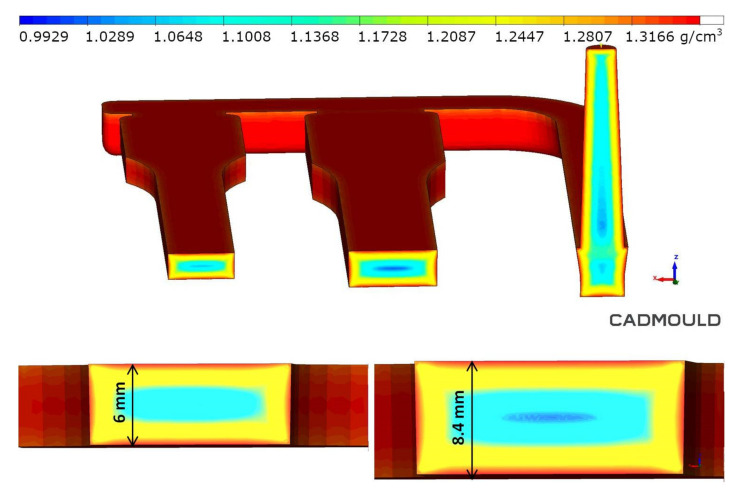
Density distribution in the measuring cross-section of 6mm and 8.4 mm thick polyamide test specimens.

Interestingly, other simulation results, e.g., samples’ density distribution calculated in the simulation correspond very well to the average density of samples obtained in real tests. (Figure 25). Density values of foamed parts marked C ranged from 1.14 to 1.28 g/cm^3^ in the transition layer.

**Figure 26:**
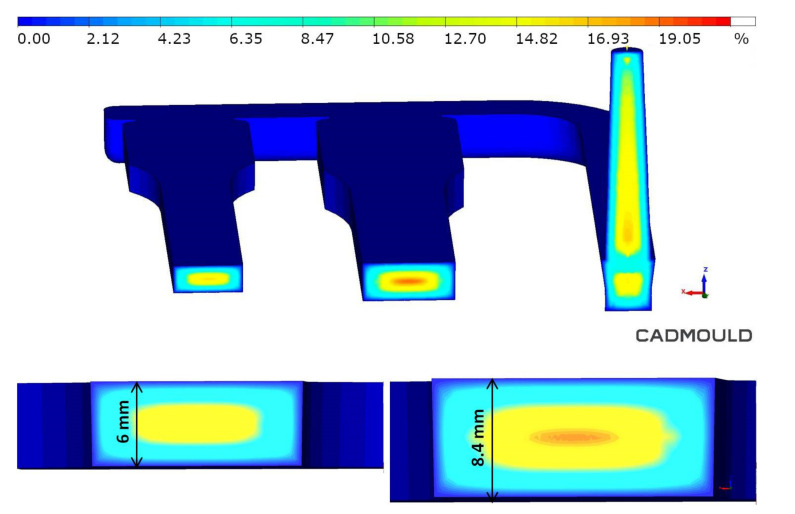
Gas volume distribution in the measuring cross-section of 6 mm and 8.4 mm thick polyamide samples.

The gas volume fraction calculated in the simulation corresponds to the weight loss obtained under real conditions. In the transition layer, which is crucial due to its mechanical properties, the gas volume fraction ranged from 4% to 8% (Figure 26).

For engineering materials, the relation between strength properties and density or specific weight is important. Therefore, the obtained results of flexural and tensile modulus in the form of normalized modulus (according to Volpe’s work, it is a product of the modulus quotient of foamed to unfoamed sample and the density quotient of unfoamed to foamed simple) were compared. It was observed that for thick-walled PA66 GF30 moldings, the normalized modulus increased to a value above 1 (Table 7). At the same time, for the Young modulus determined in the tensile test, these relationships are close to 1. For bending test, it is possible to observe a higher normalized modulus for samples with a thickness of 8.4 mm and when using higher nitrogen pressure in the microcellular injection molding process.

**Figure 27:**
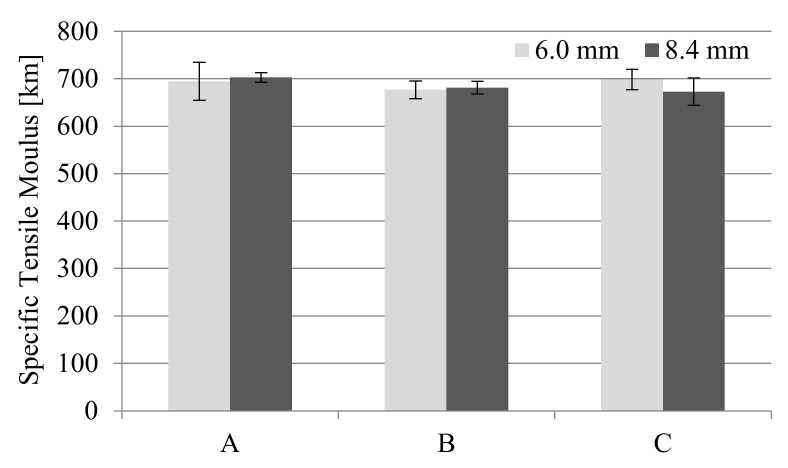
Influence of MuCell^®^ technology on specific tensile modulus of all the PA66 GF30 samples, where A is a solid sample, while B and C are foamed at 16 and 20 MPa nitrogen pressure respectively.

Relating the tensile strength and Young modulus of the moldings to their specific weight is also a source of interesting conclusions. The obtained results indicate that despite such a significant increase in the thickness of the foamed moldings, their specific modulus remained at a similar level as for the solid samples. This mainly applies to moldings with a thickness of 6 mm (Figure 27).

**Figure 28:**
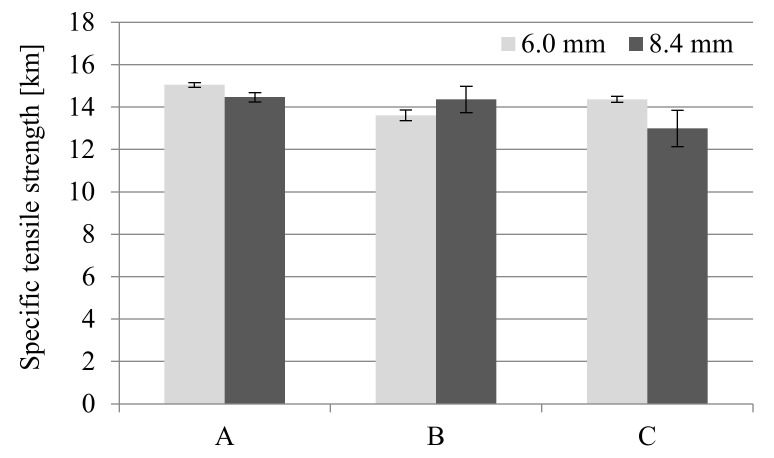
Influence of MuCell^®^ technology on specific tensile strength of all the PA66 GF30 samples, where A is a solid sample, while B and C are foamed at 16 and 20 MPa nitrogen pressure respectively.

For samples with a thickness of 6 mm, very good specific strength values can be observed, which are comparable to solid moldings (Figure 28). Even better results of specific strength relate to the data obtained in the bending test. The cell structure of foamed PA66 GF30 samples is so homogeneous and contains pores of small dimensions that the tensile test does not observe the effect of these pores of structural notches weakening the mechanical strength. This means adopting appropriate parameters of the MuCell^®^ process, thanks to which excessive cell growth in the core zone was not observed, which can be evidenced by the SEM images [22]. For comparison, the specific tensile strength and Young’s modulus for the aluminum alloy 2024-T6 are 14.5 km and 2642.8 km, respectively.

**Figure 29:**
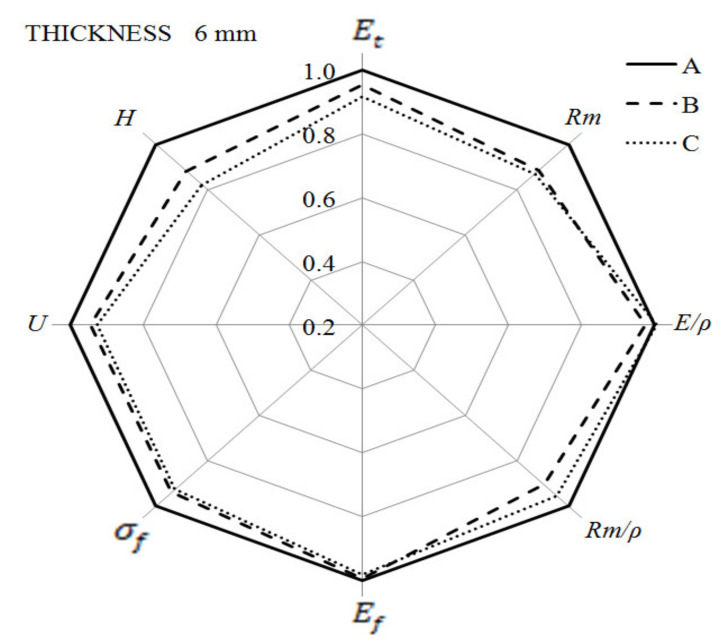
Changes in mechanical properties of 6.0 mm thick foamed samples as compared to solid PA66 GF30 moldings.

**Figure 30:**
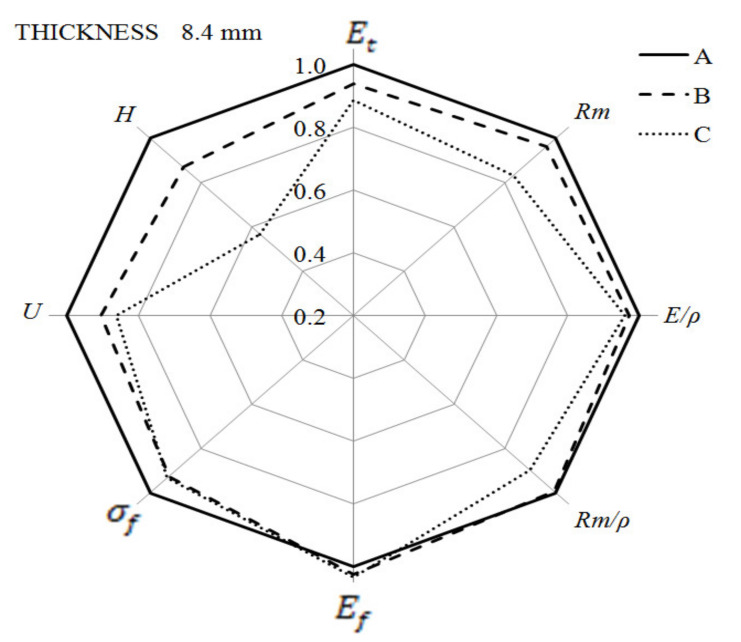
Changes in mechanical properties of 8.4 mm-thick foamed samples as compared to solid PA66 GF30 moldings.

The summary of the presented results of the mechanical properties are two radar charts (Figure 29 and Figure 30). The results obtained for solid samples were adopted as the reference level (marked as 100%). For both types of samples, their significant thickness reduces the hardness, especially for the thicker ones, obtained with the use of higher gas pressure. The impact strength of the foamed samples is lower, but in comparison to the solid moldings, the decrease does not exceed 15%. The most interesting part of the analysis concerns the comparison of the strength properties obtained for all specimens in static tensile, bending and impact tests. From the strength characteristics tested, there is a general tendency to decrease the value for moldings made with the MuCell^®^ technology, but not more than 20% to solid samples.

The exception is the bending modulus, the value of which for foamed 8.4 mm thick samples is higher than for solid specimens. Porous materials show even better results after relating their modulus value to the density. Foamed samples are also characterized by very good flexural strength values, only a few percentages worse than the PA66 GF30 solid composite. It can be concluded that the multilayer structure, fiber orientation, and homogeneous pore distribution are the keys to obtaining the PA66 GF30 composite with a lower density. Even though it is an obvious conclusion, such a result is difficult to be realized. In this case, the gas pores are not micro notches in the structure of thick-walled moldings and have no significant influence on the decrease of composite mechanical properties. Good strength properties of porous PA66GF30 samples result from easier fibers orientation in the polyamide matrix with reduced viscosity, which was confirmed in an earlier article [22].

## 4. Summary

It can be concluded that the good mechanical properties of thick-walled PA66 GF30 moldings, obtained in the microcellular injection molding process strongly depend on the sample morphology, mainly on the distribution of fibers and cells in the sandwich structure. In the cross-section of foamed moldings, location and shape skin layer, transition and core zone have anisotropic structure. It has been confirmed that is possible to obtain no defects, thick-walled PA66 GF30 molded parts with the use of the MIM by the MuCell^®^ method. Samples were free from warpage or sink marks, although the holding pressure was set to 0.3 seconds only. Nitrogen pressure significantly affects the mechanical properties and density of the PA66 GF30 molded parts. Despite the considerable thickness of moldings and high pressure of the nitrogen, a fine-cell structure with regular pores (cells) of about 20–30 µm was obtained, contrary to twice as large [14,20] or similar pores [13] in other papers. The appropriate fiber orientation, documented in an earlier article [22] and confirmed by additional SEM and FEM analysis, is also of great importance for the obtained properties. Therefore a slight decrease in mechanical properties was observed, which is in agreement with other researches [12,14,17]. It has been shown that the MuCell^®^ technology enables the manufacturing of thick-walled polyamide moldings with strength properties similar to those of aluminum alloys. The research results may contribute to the increased application of reinforced polyamide in advanced lightweight structures. FEM tests can help analyze the microporous injection molding process, but with thick-walled moldings, it requires further testing using mesh-based programs, such as Moldex 3D. Weight reduction is an additional significant effect, which, combined with mechanical properties (specific strength), places these composites among important engineering materials.

## Figures and Tables

**Table 1:** Process parameters used during parts production in standard injection molding and MuCell^®^ technology.

Parameters	Standard Injection Molding	MuCell^®^ Technology
filling pressure	58 MPa	70 MPa
switching point *	20 mm	1.0 mm
melt temperature	285 °C	285 °C
holding pressure	28.5 MPa	14 MPa
holding time	7 s	0.3 s
mold temperature	90 °C	90 °C
cooling time	30 s	50 s

* switching point between filling and holding phase.

**Table 2:** Gas parameters used during foam parts production.

Type of Sample	Gas Parameters Used in the MuCell^®^ Technology	
	Pressure, MPa	Flow Rate, kg·h^−1^
A *	-	-
**B**	16	0.36
**C**	20	0.36

* standard injection molding process.

**Table 3:** FEM simulation parameters of MIM process.

FEM Simulation Parameters	
Mesh type	Irregular triangular
Element size	1.78 mm (0.5%)
Quantity node	80,731
Model surface	74,067 mm^2^
Elements volume	214,509 mm^3^

**Table 4:** Thickness (in mm) of a skin transition and core layer together with its percentage in a total sample’s thickness based on SEM images after scanning sample cross-section from left to right sample edge.

Sample *		Skin (Left Side)	Trans (Left Side)	Core	Trans (Right Side)	Skin (Right Side)
C8	Thickness [mm] Percentage [%]	1.32 6.78	3.89 19.97	9.29 47.65	3.89 19.97	1.10 5.63
C6	Thickness [mm] Percentage [%]	0.77 5.31	4.87 33.59	4.40 30.34	3.73 25.72	0.73 5.03

* coefficient of variation for all measured zones does not exceed 5%.

**Table 5:** The enthalpy of melting (ΔH_m_) of PA66 composite filled with 30 wt% glass fiber; melting temperature (T_m_) of the composite; sample mass and calculated crystallinity (α) of 6 mm (A6. B6. C6) and 8.4 mm (A8. B8. C8) thick molded pieces.

Sample	ΔHm [J/g]	ΔHm Std dev. [J/g]	Tm [°C]	Sample Mass [mg]:	α [%]
A6	−39.97	0.89	262.37	7.81	30.37
B6	−42.12	0.49	266.80	7.80	32.00
C6	−40.77	0.13	263.10	8.10	30.98
A8	−31.25	1.54	263.80	11.43	23.74
B8	−35.26	0.79	264.93	11.85	26.79
C8	−33.78	1.46	264.57	8.91	25.67

**Table 6:** Influence of MuCell^®^ technology on the density of all the PA66 GF30 samples.

Thickness mm	Density kg·m^−3^	Type of Sample		
		A	B	C
6.0 mm	average value	1344.6	1287.8	1261.8
	standard deviation	7.0	5.6	22.9
	coefficient of variation, %	0.52	0.44	1.80
8.4 mm	average value	1337.8	1302.9	1243.8
	standard deviation	32.9	7.9	13.8
	coefficient of variation, %	2.46	0.61	1.11

**Table 7:** Influence of MuCell^®^ technology on normalized modulus^*^ of all the PA66 GF30 samples.

Thickness mm	Density kg·m^−3^	Type of Sample		
		A	B	C
6.0 mm	bending test	1.00	1.04	1.05
	tensile test	1.00	0.99	0.98
8.4 mm	bending test	1.00	1.05	1.12
	tensile test	1.00	0.97	0.95

^*^ True comparison between the samples with different densities, the flexural modulus was normalized according to Equation, Volpe et al.—see [14].

## Data Availability

Not applicable.
